# Electrolyzers-HSI: Close-Range Multi-Scene Hyperspectral Imaging Benchmark Dataset

**DOI:** 10.1038/s41597-025-06279-9

**Published:** 2025-11-19

**Authors:** Elias Arbash, Ahmed Jamal Afifi, Ymane Belahsen, Margret Fuchs, Pedram Ghamisi, Paul Scheunders, Richard Gloaguen

**Affiliations:** 1 Helmholtz-Zentrum Dresden-Rossendorf (HZDR) - Helmholtz Institute Freiberg for Resource Technology (HIF), Freiberg, Germany; 2 University of Antwerp, Antwerp, Belgium; 3 National School of Applied Sciences of Oujda, Oujda, Morocco

**Keywords:** Imaging techniques, Computer science, Near-infrared spectroscopy, Scientific data, Imaging and sensing

## Abstract

The global challenge of sustainable recycling demands automated, fast, and accurate material detection systems that act as a bedrock for a circular economy. Integrating front-tier technologies into advanced recycling systems democratizes access to AI-driven sustainability, and transforms waste analysis from isolated research efforts into real-time, scalable industrial practice. This integration not only accelerates material recovery but also strengthens the technological backbone required to achieve large-scale recycling and alignment with the Green Deal ambitions. In response, we introduce **Electrolyzers-HSI**, a new multimodal benchmark dataset designed to accelerate the recovery of critical raw materials through accurate electrolyzer materials classification. The dataset comprises 55 co-registered high-resolution RGB images and hyperspectral imaging (HSI) data cubes spanning the 400–2500 nm spectral range. This enables non-invasive analysis of shredded electrolyzer samples, facilitating quantitative material classification. We evaluate various analytical methods, including state-of-the-art (SOTA) Transformer-based deep learning (DL) architectures, to validate the dataset for robust electrolyzers identification. The openly accessible dataset and codebase promote reproducible research and facilitate broader adoption of smart and sustainable E-waste recycling.

## Background and Summary

Hydrogen technology, particularly electrolyzers, receives focused attention due to its role in the energy transition strategy as a solution for energy transport and storage. Research and development put strong efforts into increasing the efficiency and upscaling of the main electrolyzer types, providing productive momentum to develop recycling strategies in parallel. The recovery of the valuable and critical resources contained in electrolyzers will contribute to securing electrolyzer raw material cycles, which support the sustainability of hydrogen-related strategies^1^.

Electrolyzers’ recycling can benefit from HSI sensor technology along with SOTA ML and DL data processing models to precisely identify and recover critical materials, enhancing resource efficiency and circularity. HSI sensors acquire detailed spectral information across hundreds of spectral bands, each encoding unique interactions between the incident light and the material properties^2^. The non-invasive capability of HSI to acquire spectral characteristics from the scanned surface enables precise identification of different materials based on their spectral signatures. Apart from remote sensing applications, e.g., earth observation^3,4^, HSI offers essential capabilities for close-range sensing applications such as agriculture^5^, food^6^, healthcare^7^ and industry^8^.

Transformer-based DL models^9^ have become a cornerstone of SOTA data processing methodologies, excelling in real-time performance and high accuracy across diverse domains, including natural language processing with large language models such as ChatGPT^10,11^ and computer vision, including both images^12^ and video^13^. Recycling applications demand accurate, rapid and dynamic solutions that greatly benefit from the application of HSI with these SOTA processing modalities that have end-to-end feature extraction capabilities when training data is abundant. This allows the detection of spectral features and patterns that can reveal unique material characteristics, supporting decision-making in recycling facilities.

RGB images have high spatial resolution, revealing fine surface details, and their precise spatial features (e.g., traditional morphological features) provide clarified appearance-based characteristics for automated sorting in recycling streams. However, they may lack reliability for material identification due to appearance variations in the samples’ end-of-life conditions. In contrast, material spectral features, derived from high spectral resolution HSI, offer a more robust criterion for accurate material identification and classification. In this context, in-line, non-invasive scanning routines that combine high spatial resolution RGB with high spectral resolution HSI data emerge as a powerful solution. This multimodal approach not only enables the extraction of rich appearance features, that can further support precise point-wise validation but also captures detailed spectral characteristics essential for material-wise investigation. When integrated within ground-breaking processing frameworks, these complementary modalities significantly boost detection performance, surpassing what can be achieved with either modality alone, and lay a strong foundation for reliable, scalable industrial recycling systems. This aligns with sustainable development and the circular economy, which seeks to enhance resource recovery, reduce waste generation, and supports global sustainability goal 12: Responsible Consumption and Production^14^ through recycling^15,16^.

In this study, we contribute to sustainability by optimizing the decision-making routines in E-waste recycling streams for electrolyzers materials. We selected high-temperature electrolyzers (HTEL) because their components contain a range of high-tech and critical raw materials, i.e., rare-earth elements, Ni, Zr, and Mn contained in the Anode and Cathode ceramics of the HTEL, as well as relevant metals (frame, interconnectors, meshes). Accordingly, HTEL represents a valuable source of secondary raw materials. Our core objective is to integrate the emerging SOTA data processing methodologies for achieving accurate and fast detection of major components in electrolyzers’ recycling streams using non-invasive sensors to advance the decision-making of downstream E-waste recycling. For this reason, we provide a new multi-scene, multi-modality, high-resolution benchmark dataset of electrolyzers materials and investigate the performance of the native Transformer-based HSI processing models for the identification of electrolyzers materials. Additionally, we identify performance bottlenecks and highlight further optimization directions.

Multi-scene HSI benchmark datasets are crucial for the development of DL models to ensure their generalization across diverse scenes, since such datasets mimic industrial applications with continuous data acquisition (new HSI scenes) of mixed sample streams, over moving conveyor belts. High-quality and standardized datasets help models to learn robust and transferable representations, reducing the risk of overfitting. By exposing a model to numerous scenes and samples, it captures universal patterns and features inherent to the materials regardless of the different scenes. This directly improves its adaptability and reliable performance across multiple domains and applications. The Electrolyzers-HSI dataset was developed to address the complete absence of publicly available imaging datasets, either RGB or hyperspectral, focused on electrolyzer components, particularly in their end-of-life state. Our contribution in this work can be summarized as follows:

- Introducing Electrolyzers-HSI: electrolyzers components imaging dataset comprising 55 high spectral resolution HSI data cubes acquired in the visible-near infrared (VNIR) and shortwave infrared (SWIR) ranges, each paired with their high spatial resolution co-registered RGB twin image and classification ground truth masks.
- Validating the dataset usage for electrolyzers identification with multiple standard ML and DL single- and multimodal Transformer-based models.
- Extending pixel-level HSI analysis toward object-level sorting via zero-shot segmentation for background masking and foreground processing by combining RGB-HSI features in hierarchical fusion. This enables automated detection suited for shredded recycling streams.
- Identifying application limitations and performance bottlenecks of Transformer-based architectures applied in close-range HSI applications, exposing the constraints hindering scalability. This diagnosis informs the technical development of future models tailored to material classification tasks.
- Releasing complete inference code, model weights, and application pipelines, ensuring reproducibility and practical deployment in recycling systems.

**Fig. 1:**
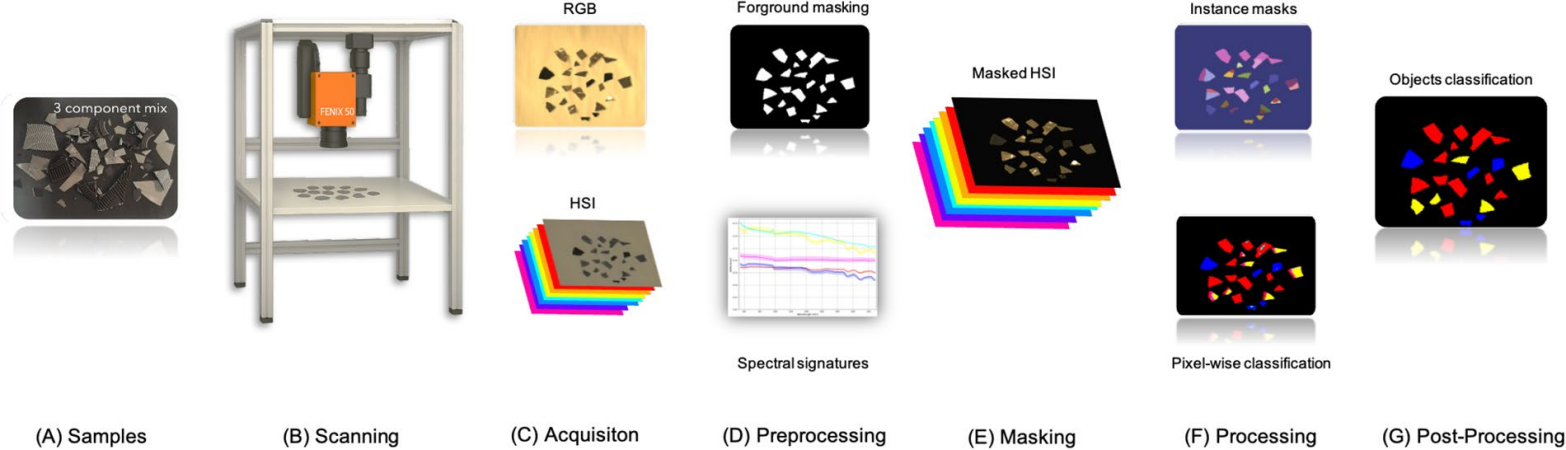
An overview of Electrolyzers-HSI dataset acquisition and processing pipeline **(A)** The fragments of the shredded electrolyzer cells were organized into controlled scenes containing up to five material classes. **(B)** Each sample configuration was scanned using a dual-modality setup composed of high-resolution RGB and HSI sensors (400–2500 nm), capturing refined spatial and rich spectral features. **(C)** The RGB images and HSI data cubes are acquired and coregistered. **(D)** The parallel preprocessing pipelines of the two modalities: dimensionality reduction and normalization of HSI data cubes and zero-shot segmentation of the foreground objects in the RGB images. **(E)** HSI data background masking and foreground processing. **(F)** Pixel-wise classification on the masked HSI and instance masks projection for majority voting. **(G)** Object-wise electrolyzers classification.

Figure 1 presents an overview of the Electrolyzers-HSI workflow from sample preparation to the final object classification. By addressing material-specific detection under realistic conditions, Electrolyzers-HSI supports the development of automated, material-aware recycling pipelines, going beyond the scope and limitations of existing datasets.

### Previous Work

With hundreds of spectral bands per pixel, HSI suffers from the curse of dimensionality and high redundancy. This complexity necessitates advanced, non-linear operations to effectively process the data, as traditional linear methods struggle to capture the intricate patterns. DL models, with their non-linear architectures, are well-suited for this task as they can uncover complex relationships within the data. As a consequence, they excel in extracting joint spatial-spectral features, making them ideal for HSI data processing^4^. Accordingly, several DL modalities were applied for HSI processing and classification, including fully connected networks^17^, recurrent neural networks^18^, convolutional neural networks (CNN)^19,20^ and Transformers^21,22^. Hybrid implementations of Transformers with other techniques like CNN were utilized^23,24^ to leverage the convolution mechanism for local spatial feature extraction together with the self-attention mechanism of Transformers, which enables the capture of both short- and long-range dependencies when plentiful data is provided. Following the trend in developing HSI processing models, we focus on pure Transformer-based models, in particular the original implementation of Transformers in HSI^21^, avoiding further advanced variants like^25,26^ in order to highlight the bottlenecks and performance limitations of the original architecture.

Data availability and quality are crucial for developing effective DL processing methods. However, HSI datasets for E-waste remain limited to only a few studies. Table 1 provides an overview of the referenced datasets, including the number of HSI scenes or data units reported by the authors, the available modalities, sensor names, spectral ranges, and the specific research objectives. Picon *et al*.^27^ provided Tecnalia WEEE, a 13-scene HSI dataset for the detection of different metallic E-waste samples, scanned in the VNIR. Bonifazi *et al*.^28^ explored SWIR HSI for polymer characterization to enhance plastic identification, aiding quality control and sorting processes in E-waste recycling streams. Using point measurement devices, Leone *et al*.^29^ acquired a plastic hyperspectral reflectance dataset in the VNIR and SWIR spectral ranges from various samples, including pristine and degraded specimens. Gathering and utilizing up to 9 HSI data cubes from the HSI scenes in^30,31^, Arbash *et al*.^32^ investigated the performance of SOTA HSI classification models in polymer classification. Thermal E-Waste by Paulraj *et al*.^33^ is a thermal HSI dataset in the longwave infrared (LWIR) range for the classification of mixed E-waste samples (metals, plastics, PCBs, glass). Aiming at predicting the color of regranulates based on the color content of input flakes, Lambers *et al*.^34^ generated a hyperspectral dataset comprised of 185 measurements of 37 samples: 29 flakes and 8 colored samples. Several works demonstrate the value of multimodal RGB-HSI data for enhanced material characterization and sorting. PCB-Vision by Arbash *et al*.^35^ is a dataset of 53 HSI-RGB scenes of printed circuit boards (PCBs) with segmentation ground truth for the PCB board and several main PCB components, including integrated circuits, capacitors and connectors. Casao *et al*.^36^ contributed with Spectral Waste, an RGB-HSI dataset containing 852 non-overlapping labeled and 6803 unlabeled scenes from an operational plastic waste sorting facility. The authors proposed a processing pipeline, integrating different DL modalities for general waste object segmentation, along with a co-registration method for the different modalities. De Lima Riberio *et al*.^30^ characterized the improvements of polymer identification by combining Raman point measurements with HSI.

**Table 1:** Overview of HSI E-waste datasets.

Dataset	Data	Modality	Range	Sensor	Task(s)
Leone *et al*.^29^	+108 Point measurement	Hyperspectral vectors	350–2500 nm	FieldSpec 4 spectroradiometer	Polymers classification
Tecnalia WEEE^27^	13 scenes	HSI	400–1000 nm	Specim PHF Fast10 camera	E-waste metals segmentation
WEEE Plastic^28^	Multiple plastic fragments scenes	HSI	1000–2500 nm	Specim ImSpector N25E	Polymers classification
Thermal E-Waste^33^	Multiple E-waste IR scenes	HSI	8000–15000 nm	FLIR ORION SC7000	E-waste samples classification
Lambers *et al*.^34^	37 samples	HSI	400–1000 nm	Innospec GreenEye	Color prediction of regranulate
Polymers^32^	9 scenes	HSI	380–2500 nm	Specim FENIX	Polymers classification
SpectralWaste^36^	852 labelled scene + 6803 unlabelled scenes	RGB + HSI	1000–1700 nm	Teledyne DALSA Linea + Specim FX17	E-waste samples segmentation
PCB-Vision^35^	53 scenes	RGB + HSI	400–1000 nm	Teledyne Dalsa C4020 + Specim FX10	PCB components segmentation
Electrolyzers-HSI	55 scenes	RGB + HSI	380–2500 nm	Teledyne Dalsa C4020 + Specim FENIX	Electrolyzers classification
De Lima Ribeiro *et al*.^30^	23 samples + 1 scene	Raman + HSI	400–3400 cm^−1^, 480–5300 nm	HORIBA ARAMIS Raman spectrometer, FENIX + FX50	Polymers identification

As summarized in Table 1, existing HSI datasets for recycling are limited to single modalities or narrow spectral ranges and largely focus on mixed e-waste or municipal solid waste segmentation. Electrolyzers-HSI introduces the first dataset dedicated to electrolyzer components, addressing a critical gap in recycling research. It combines pristine and dismantled end-of-life samples to capture realistic degradation and contamination, spans the full 400–2500 nm range beyond VNIR- or SWIR-only datasets, and co-registers hyperspectral with RGB imagery to enable spatial-spectral features fusion.

By targeting material-specific detection under operational conditions, Electrolyzers-HSI supports the development of automated, material-aware recycling pipelines. Its broad spectral coverage and multimodal design extend utility beyond classification, opening avenues for new methodological advances and accelerating the integration of AI-driven sustainability into industrial practice.

## Methods

**Fig. 2:**
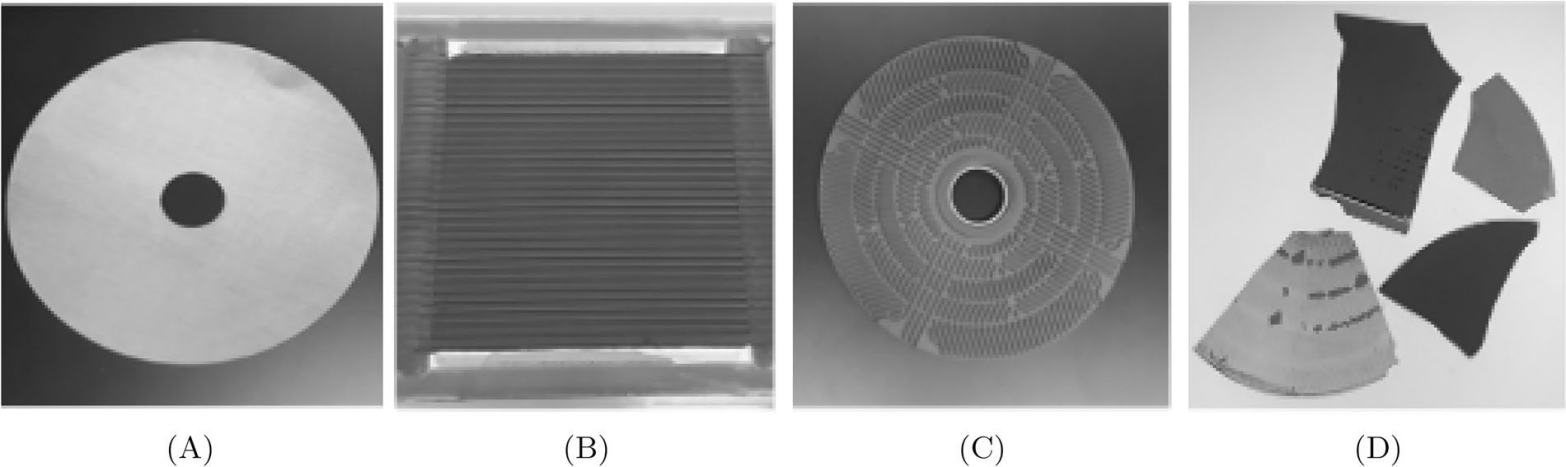
Dataset samples: **(A)** Ni-Mesh - **(B)** HTEL - **(C)** Steel - **(D)** Samples from mixed origins and states.

The five materials included in the Electrolyzers-HSI dataset represent the core functional components of HTEL systems, selected for their industrial importance and direct relevance to automated recycling scenarios. Our samples comprise shredded pieces from HTEL ceramic cells, Ni-mesh, and interconnector steel plates^1^ sourced in both new and end-of-life states (Fig. 2). The ceramic layers expose two functionally distinct surfaces, the anode and cathode, each with different chemical compositions and spectral signatures critical to the operation and recovery of high-value compounds such as rare earth elements (REEs). Ni-mesh, the primary current distributor and electrode support, is widely used across electrolyzer designs and forms a key target for nickel reclamation. Interconnector steel plates likewise present two functional faces: untreated “steel-Gray” and oxidized “steel-Black,” both common metallic fractions in electrolyzer stacks, whose accurate separation is essential for generating clean steel streams in metallurgical recycling. Together, these five classes, Mesh, Steel-Gray, Steel-Black, HTEL-Anode, and HTEL-Cathode, capture the expected surfaces exposed to imaging sensors in electrolyzer recycling streams.

Unlike broader e-waste datasets that typically distinguish only coarse object categories, e.g., plastics vs. metals, Electrolyzers-HSI enables material-level discrimination between visually similar but functionally distinct electrolyzer components. This targeted scope maximizes recovery value, ensures efficient downstream processing, and directly addresses the material-specific challenges of recycling technologies vital to the green hydrogen economy.

The HTEL stack cells were physically shredded to simulate real-world recycling conditions, and the resulting material fragments were then systematically mixed to generate samples containing different numbers of material classes: 6 samples containing only a single class, 31 samples containing two classes, 9 samples with three classes, 4 samples with four classes, and 5 samples with five classes. This structured approach enables both controlled single-class learning and multi-class classification. The scenes provide controlled single- and multi-material environments for bias detection and model validation, allowing class-specific performance to be isolated and compared against mixed-class scenarios. This design makes it possible to assess how contextual complexity influences generalization and helps reveal potential biases in learned spectral representations. Each side of the material surface (front and back) was scanned, ensuring comprehensive spectral coverage for all targeted classes.

The scanning device is an AisaFENIX (Specim, Spectral Imaging Ltd.) push-broom HSI camera covering the VNIR-SWIR wavelength range [400–2500 nm] with 450 bands (spectral resolution: VNIR 3.5 nm, SWIR 12 nm). The detector records 384 pixels per line, while image length is defined by the scanning speed and integration time. In parallel, high-resolution RGB data were acquired using an LT-400 CL 3 CMOS line-scan camera. The cameras are operated in the SisuRock workstation (Specim, Oulu, Finland) with optimized fixed focus distances, providing a pixel size of 1 mm at the scan plane. The main acquisition parameters are summarized in Table 2.

**Table 2:** The parameters of the RGB and HSI sensor used for the acquisition.

Parameters	LT-400 CL 3 CMOS	Aisa FENIX
**Sensor type**	RGB CMOS	Push-broom
**Spectral range**	RGB	400–2500 nm
**Spatial resolution**	4096 px/line	384 px/line
**Pixel size**	0.16 mm	1.6 mm
**Bands**	3	VNIR: 175—SWIR: 275
**Line rate**	16 KHz	100 Hz

**Fig. 3:**
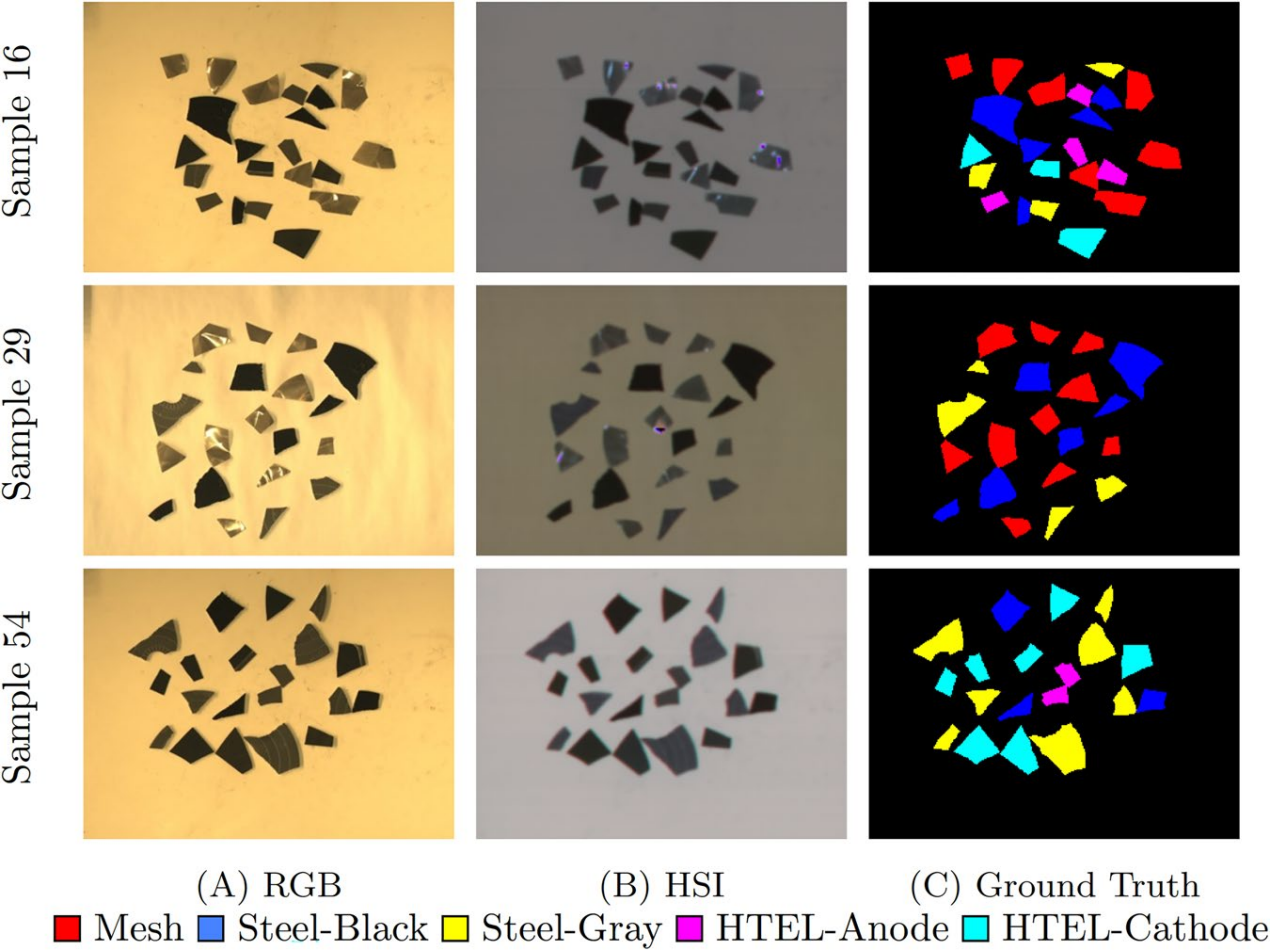
Three scan samples, each consisting of a high spatial resolution RGB image, a HSI data cube (false color representation, using bands 2069 nm, 1792 nm, and 1401 nm) and the ground truth.

Each scanning session begins with a dark (closed shutter) acquisition for sensor noise corrections and an acquisition of a white reference panel (Zenith Polymer Diffuse Reflectance Standard  >99%) for radiance to reflectance conversion. Calibration images containing six fiducial markers are used to determine camera offsets and compute affine transformations, ensuring accurate co-registration of the HSI and RGB data products. Figure 3 visualizes scans of samples 16, 29, and 54, consisting of the RGB images, false color representation of the HSI [2069, 1792, 1401] nm, and the corresponding ground truth masks. Compared to the true color visualizations that yield dark tones and low inter-class separability, the selected false color bands enhance visual contrast between materials by exploiting the spectral differences in the SWIR region. The RGB image provides high-resolution spatial features that support classification. However, since the samples’ shape can differ depending on the end-of-life status, spectral features are the main classification key.

**Fig. 4:**
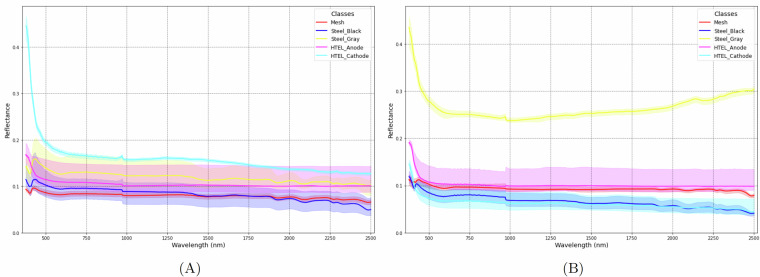
The spectral signatures of the five classes for the training set (A) and the test set (B).

Figure 4 shows the average spectral signatures of the five classes used as model inputs for both the training set (A) and test set (B). The dataset presents a particularly challenging case for spectral feature learning: dark-colored surfaces such as Steel-Black, HTEL Anode, and Mesh produce low-reflectance signals across the entire VNIR-SWIR range, and share similar dark or gray tones, making color cues non-distinctive. Moreover, further differences appear between the two sets, e.g., spectra of Steel-Gray are more distinct in the test set, while the training and test spectra overlap in case of Mesh and Steel-Black. Such variations reflect differences in sample degradation and surface properties across scans, further challenging model generalization. These factors establish Electrolyzers-HSI as a benchmark dataset for testing model robustness under adverse sensing conditions.

Figure 4 reveals distortions of both training and test spectra at the extremes (<450 nm and >2450 nm). To obtain efficient and reliable data processing, the first 50 and last 40 bands of the 450 acquired bands were discarded, resulting in 360 usable spectral channels. This exclusion corresponds to the detector’s range limits, where sensor sensitivity drops and signal uncertainties dominate. Removing these bands is a standard procedure to avoid regions with little or no coherent information. The retained wavelength range provides smoother, more coherent reflectance profiles that form a reliable basis for analysis and model training.

Following this, spectral binning was applied by averaging every two adjacent bands, reducing the dimensionality from 360 to 180 bands. This strategy was chosen because contiguous HSI bands are highly correlated, and no strong unique absorption features were observed across the full 400–2500 nm range. Pairwise averaging minimizes redundancy while preserving local spectral trends, improves the feature-to-sample ratio, thereby stabilizing convergence and reducing computational load without erasing discriminative information. Such dimensionality management is particularly important for DL models with limited labeled data: excessive redundancy can lead to overfitting, especially in transformer-based architectures with many parameters.

Table 3 summarizes the preprocessing steps of the HSI and RGB data to ensure spectral fidelity, spatial alignment, noise minimization, and establishment of post-processing object-level classification. Raw data from all sensors were processed through a real-time preprocessing pipeline powered by Crunchy^37^, an in-house Python-based engine designed for high-throughput hyperspectral data handling. Original HSI scenes are of size  > 400 Mb per data cube and comprise approximately 640 × 1517 × 450 pixels, while RGB images measure 3840 × 9102 × 3 pixels. Post co-registration, images were cropped to 240 × 325 pixels to center the shredded electrolyzer fragments and exclude redundant background. This ensures the content is concentrated on the materials of interest, while reducing data volume for more efficient memory handling and computation. Ground-truth masks of electrolyzer components were manually created for supervised training and validation. Moreover, vector-wise normalization adjusts reflectance values from shiny metal surfaces that exceed 1.0, enhancing spectral stability and model convergence. Finally, RGB foreground segmentation was performed using zero-shot object detection and segmentation modalities for subsequent removal of the undesired background and isolation of foreground vectors.

**Table 3:** The data preprocessing steps of the HSI and RGB data of Electrolyzers-HSI.

Step	Procedure	Purpose
**Lens distortion**	Optical correction using calibrated camera parameters.	Removes geometric distortion.
**Noise removal and reflectance conversion**	Dark current subtraction and reflectance conversion, using a closed shutter and a white reference panel acquisition: $\frac{{I}_{raw}-{I}_{dark}}{{I}_{white}-{I}_{dark}}$	suppresses sensor noise, converts raw radiance to reflectance.
**Co-registration**	Six ArUco markers used to compute the affine transformation.	Ensures pixel-level multimodal alignment.
**Cropping**	Non-material regions removed post-registration.	Reduces data size, isolates fragments.
**Ground truth**	Using ’LabelMe’ annotation tool to create ground truth masks.	Training ground truth.
**Spectral normalization**	Vector-wise normalization applied post-calibration to handle reflectance > 1.0 (metal glare).	Stabilizes spectra, aids ML model convergence.
**RGB segmentation**	Zero-shot modalities for RGB instance prediction.	Background removal and foreground preserving of HSI.

## Data Record

Electrolyzers-HSI^38^ is available at: “https://rodare.hzdr.de/record/3668”. The dataset contains a total of 55 triplets of images consisting of co-registered high-resolution RGB images and their high spectral resolution HSI twins, in addition to the ground truth masks. Out of 4,290,000 pixel vectors, a total of 424,169 labeled pixels are generated from the ground truth masks.

The dataset is structured into numerically labeled subfolders, each corresponding to a sample. Within each subfolder, four files are provided: an RGB image in ‘.jpg’ format, a ground truth segmentation mask in ‘.png’ format, a hyperspectral image stored in ‘.img’ format, and the associated header file in ‘.hdr’ format containing metadata necessary for interpreting the hyperspectral data. This consistent folder structure ensures straightforward access and alignment between the RGB, ground truth, and hyperspectral modalities for each sample.

**Fig. 5:**
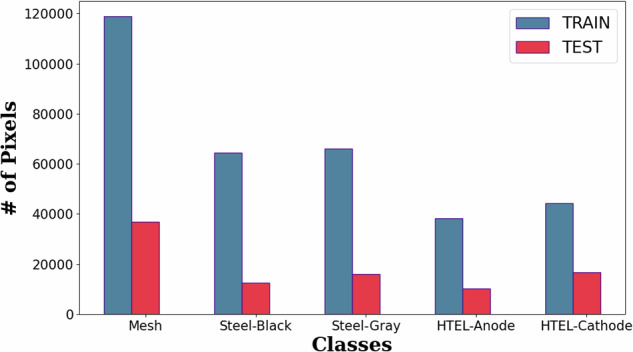
Bar plot comparing the number of training and test pixels per class.

From the entire dataset, 44 images were used for training and 11 for testing, following a carefully designed split aimed at minimizing overlap between samples from the same physical components. This reduces the correlation of the same origin material and allows unbiased model evaluation. In total, 336,215 pixels were used for training and 87,954 for testing. Figure 5 presents the sizes of the training (blue) and test (red) sets across the five classes. As shown, the dataset is notably imbalanced, with the “Mesh” class comprising 35.9% of the training samples, substantially more than the other classes, which are more evenly distributed. If unaddressed, such a class imbalance can lead to biased models with poor generalization on underrepresented classes. To mitigate this issue, we applied a weighted cross-entropy loss, where class weights are computed inversely proportional to their frequencies in the training set. This approach increases the influence of minority classes during training, encouraging the model to learn more representative and invariant features across all training data.

## Technical Validation

To validate the suitability of the dataset for downstream sorting tasks in the context of electrolyzer material analysis, we implemented several ML processing pipelines for pixel-wise and object-wise electrolyzer material classification. As illustrated in Fig. 1, following the RGB and HSI data acquisition, each modality was preprocessed in parallel. HSI preprocessing involves reflectance conversion and normalization. RGB preprocessing includes the application of the pre-trained Segment Anything Model (SAM)^39^ and Grounding Dino^40^ using the method in^41^ to segment foreground objects from the background. The obtained objects’ instance segmentation masks are then used to generate masked HSI data cubes, enabling processing models to focus exclusively on pixels containing object materials only. The preprocessed data is used for training the ML and the Transformer-based modalities for pixel-wise electrolyzer classification. Finally, the instance masks are overlaid on top of the pixel-wise classification maps, and object-wise classification is achieved through majority voting within object polygons defined by the instance masks generated using the zero-shot models.

### SOTA Models

We investigated the application of electrolyzers classification using the provided dataset with several representative ML and pioneering Transformer-based^42^ HSI classification models. Transformers became the backbone of all SOTA data processing models due to their core computation process, i.e., self-attention, which serves as a suitable mechanism for detecting the spectral features when the data is properly tokenized. In the spectral domain, spectral fingerprints represent unique changes in the spectral signature at specific wavelengths, which characterize the light absorption features of the material. Spectral features are encoded in the HSI data using two variables: the reflectance value and its wavelength position in the spectrum. To effectively detect these patterns, a computational framework is required to model the relationships and affinities between its input tokens, which encode these two dimensions. The self-attention mechanism within Transformer architectures enables this by dynamically updating the representation of each token based on contextual information from all other tokens. This makes Transformers particularly suitable for extracting and modeling complex spectral features in HSI data.

Rather than more recent, specialized variants, we selected Transformer models that were among the first to be applied to images, including RGB and HSI, since they are suitable for exposing fundamental challenges and limitations of the modality.

The evaluated models are:

- Traditional machine learning (ML) models, including Random Forest, K-Nearest Neighbors (KNN), and Support Vector Machine (SVM) were employed to process individual hyperspectral vectors. Given the computational demands associated with processing large volumes of high-dimensional HSI data, these models were implemented using Dask, a parallel computing library. By leveraging Dask arrays and pipelines with a chunk size of 10,000, we efficiently distributed the computational workload, significantly reducing both memory usage and processing time.
- **Vision Transformer** (ViT) is the Transformer-encoder architecture that introduced the adaptation of Transformers^9^ to image classification tasks^43^. In its original implementation, an input RGB image is divided into non-overlapping 16 × 16 patches along its spatial dimensions. For deploying ViT on HSI, we extract spectral patches from the same spatial location to predict the class label of the center pixel. Each patch from a different band is then linearly embedded and treated as a token. The model applies a self-attention mechanism to iteratively update these token representations by incorporating contextual information from all other tokens, enabling the network to capture global dependencies and effectively perform spectral signature understanding and pixel-wise HSI classification.
- **SpectralFormer**^21^ was one of the first models to adapt Transformer architectures specifically for HSI classification. SpectralFormer is built on the ViT framework, on top of which two architectural features tailored for HSI processing are introduced: i) groupwise spectral embedding (GSE), which enriches patch embeddings by emphasizing spectral features, ii) cross-layer adaptive fusion (CAF) to enhance the feature integration across encoder layers. SpectralFormer is implemented in two configurations: a **pixel-wise** version that classifies using only the center pixel’s spectral vector, and a **patch-wise** version that processes a full 9 × 9 spatial patch (81 vectors), incorporating both spectral and local spatial context for improved classification accuracy. A patch size of 9 × 9 was selected to balance memory efficiency with classification accuracy, while also minimizing the risk of mixed spectral signatures. Larger patches tend to include pixels from multiple classes, causing more ambiguity, thus reducing the model’s performance.
- **Multimodal Fusion Transformer (MFT)**^44^ follows the SpectralFormer adaptation of Transformer-based encoders on HSI data. It is a ViT-based neural network designed for pixel-wise classification of HSI, with architectural enhancements to integrate a second modality. Built upon a standard Vision Transformer encoder, MFT introduces modifications that allow the encoder to process another modality via the classification token (CLS). Initially, both modalities undergo feature extraction via new CNN blocks. The primary modality, HSI, is processed through a combination of 2D and 3D convolutional layers to capture spatial-spectral relationships, and is then tokenized. The secondary modality, RGB in our case, is processed through a separate CNN pathway. The RGB-derived features are then incorporated into the Transformer via the CLS token, enabling cross-modal interaction. MFT employs cross-modality attention mechanisms to facilitate fusion between HSI and RGB features. As with SpectralFormer, tokens are generated from the spectral bands at the same spatial location, preserving the integrity of pixel-wise classification while enabling multimodal learning^44^.

To improve model generalization, we applied a combination of eight spectral and spatial augmentation techniques during training. Spectral augmentations included band shifting, spectral smoothing, noise addition, scaling, and channel dropping. Spatial augmentations consisted of image rotation, translation, and flipping. These transformations were applied on the fly during training, effectively increasing the size of the dataset by a factor of eight. This dynamic augmentation strategy enabled training over 600 epochs with a reduced learning rate of 1e-7. For SpectralFormer, we adopted a groupwise spectral embedding (GSE) size of 7, consistent with the original implementation. All models were trained using the Adam optimizer with a mini-batch size of 512.

### Pixel-wise Classification Evaluations

**Fig. 6:**
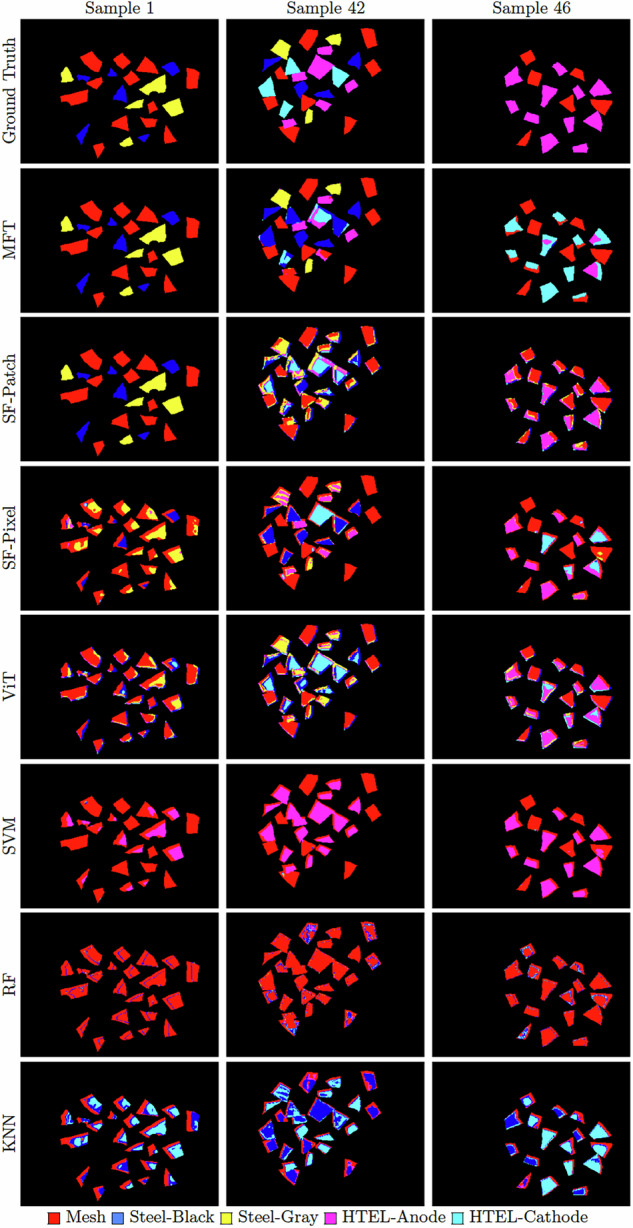
Pixel-wise classification prediction maps on samples 1, 42, and 46 from the test set.

HSI pixel-wise classification results offer valuable insights into model behavior and performance, highlighting areas for potential optimization. Figure 6 visualizes the pixel-wise prediction maps of the deployed models, and Table 4 provides the pixel-level classification performance of all evaluated models, reporting per-class F1 scores along with overall accuracy (OA) and average accuracy (AA). The results are organized from left to right: the MFT multimodality model, the single-modality SpectralFormer and ViT, and the classical ML baselines. The table indicates the following:

- The superior performance of the MFT model, which exploits both RGB and HSI modalities, is evident, achieving the highest scores across all evaluation metrics. This outcome is in line with expectations, as the high spatial resolution of the RGB images contributes significantly to class discrimination. These results highlight the power of multimodal approaches and underline the benefits of integrating complementary spatial and spectral features for more robust classification.
- The pixel-wise SpectralFormer performs second best after MFT, outperforming both the patch-wise SpectralFormer and the ViT model. This distinction provides important insights into the behavior of Transformer-based encoders when applied to HSI data. While GSE in SpectralFormer^21^ enriches token representations by integrating information from neighboring spectral bands, enriching the spectral feature, it also introduces convergence issues when the input patch contains mixed multi-class spectra, a problem illustrated in Fig. 9. As a result, the patch-wise SpectralFormer suffers from a performance drop compared to its pixel-wise counterpart. The pixel-wise variant avoids this issue by processing individual spectral vectors, allowing the Transformer to focus exclusively on the spectral features of a single material without impurities from neighboring classes. While this comes at the cost of spatial context, it guarantees spectral consistency within the token. To restore spatial awareness without compromising spectral purity, object-level context is later recovered via independent zero-shot modalities and post-processed via object-guided majority voting. This strategy provides a balance between spectral precision and spatial reasoning, ultimately improving classification robustness.
- In contrast, the ViT does not include the GSE or CAF modules from SpectralFormer. Instead, it tokenizes each spectral channel within an HSI patch as an independent input token. As a result, mixed-material signatures affect less when patches contain spectra from multiple classes compared to when patches are processed with GSE. The comparable performance between ViT and the pixel-wise SpectralFormer highlights an important drawback: while including more than one spectral vector (e.g., using the entire patch instead of just the center pixel) can improve classification by introducing richer spatial information, it also increases the risk of including mixed spectral signatures in the input, potentially leading to confusion during learning.
- The performance of the classical ML models is heavily biased toward the “Mesh” class, with significantly lower accuracy in detecting the remaining classes. This imbalance becomes even more apparent when object-wise classification is applied to the pixel-level predictions, further demonstrating the limited generalization of the models across different material types.

**Table 4:** Pixel-wise classification results for the different models in terms of the F1 score per class, overall accuracy (OA), and average accuracy (AA).

Classes	Multimodality	SpectralFormer		Transformers	Conventional Classifiers		
	MFT 9 × 9	Patch-wise 9 × 9	Pixel-wise	ViT	SVM	RF	KNN
1 (Mesh)	**86.03**	74.51	78.88	75.88	80.62	52.19	45.29
2 (Steel - Black)	**72.27**	31.39	54.67	27.42	3.26	7.41	21.67
3 (Steel - Gray)	**75.90**	32.78	52.06	45.77	15.20	14.42	6.49
4 (HTEL - Anode)	**40.68**	25.77	36.47	31.74	28.89	1.05	3.13
5 (HTEL - Cathode)	36.04	34.37	30.71	**38.48**	3.07	3.24	16.72
OA (%)	**69.30**	47.92	59.78	51.81	47.08	35.17	25.28
AA (%)	**64.29**	39.95	51.15	43.81	34.98	19.68	22.14

### Object-wise Classification Evaluations

**Fig. 7:**
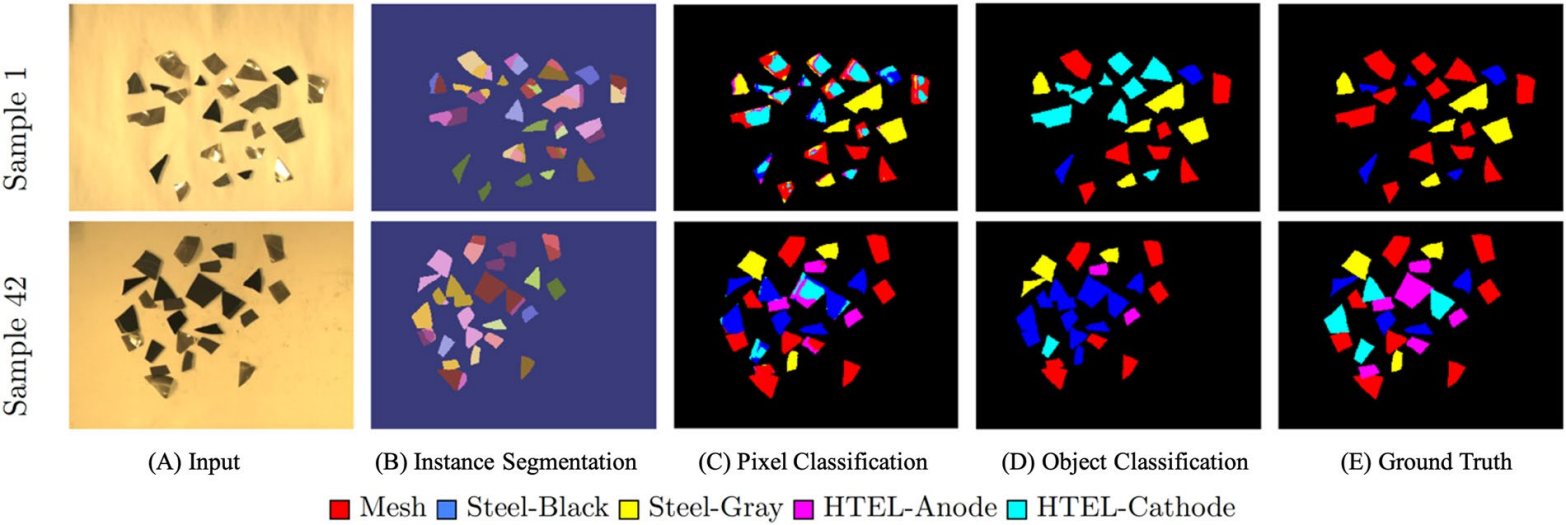
Majority voting workflow on sample 1 and sample 42: Instance masks are generated from the RGB images using zero-shot segmentation. Then, the masks are projected onto the pixel-wise classification for approximating the object-wise classification.

To better assess material detection performance, we applied an object-wise majority voting strategy based on zero-shot object segmentation. Figure 7 presents the workflow to obtain object-wise classification. Using zero-shot detection, the instance segmentation maps are generated and projected onto the pixel-wise classification for approximating the object-wise classification. In this process, objects are represented by polygons, and all pixels within the polygon are assigned the class label that is predicted most frequently among the enclosed pixels. This approach improves classification robustness by incorporating spatial neighborhood information at the object level from segmenting high-resolution RGB images. Pixel-wise predictions often exhibit noise around object boundaries, mainly due to distorted reflectance signals at the edges where light interacts with slanted or uneven surfaces. This effect is illustrated in Fig. 6, where central object regions are consistently labeled, while edge regions display higher prediction variability. The object-wise classification results are presented in Table 5. One can observe that majority voting classification consistently improves overall performance across all models when compared to their respective pixel-wise classification results. Among the evaluated classes, class Mesh was the most accurately detected, followed by Steel Black, Steel Gray, and HTEL Anode. The lowest classification performance was observed for the HTEL Cathode class. These results demonstrate the strong potential of HSI for distinguishing electrolyzers materials and also underscore the need for an extended spectral range to capture more discriminative features, particularly for materials with subtle spectral differences.

**Table 5:** Object-wise classification results via majority voting for the different models in terms of the F1 score per class, OA, and AA.

Classes	Multimodality	SpectralFormer		Transformers	Conventional Classifiers		
	MFT 9 × 9	Patch-wise 9 × 9	Pixel-wise	ViT	SVM	RF	KNN
1 (Mesh)	**99.93**	99.91	99.92	99.91	99.92	99.92	99.93
2 (Steel - Black)	82.65	76.92	82.26	**83.00**	81.59	64.71	51.63
3 (Steel - Gray)	**80.35**	41.55	73.84	58.85	1.24	3.24	27.92
4 (HTEL - Anode)	**70.19**	47.39	63.15	64.81	4.81	4.13	1.37
5 (HTEL - Cathode)	39.54	33.83	45.41	**51.40**	40.17	0.16	0.68
OA (%)	**98.16**	97.36	98.02	98.10	96.31	95.70	95.77
AA (%)	**74.55**	58.84	70.35	68.32	54.77	40.31	43.03

### Limitations & Performance Bottleneck

We highlight several factors that affect the performance of the suggested electrolyzer segmentation and classification approaches on the provided dataset. Performance drawbacks arise from object-wise segmentation based on zero-shot majority voting and computation bottlenecks for Transformer encoders when processing HSI patches, both of which necessitate improvements in tokenization and architectural design.

**Fig. 8:**
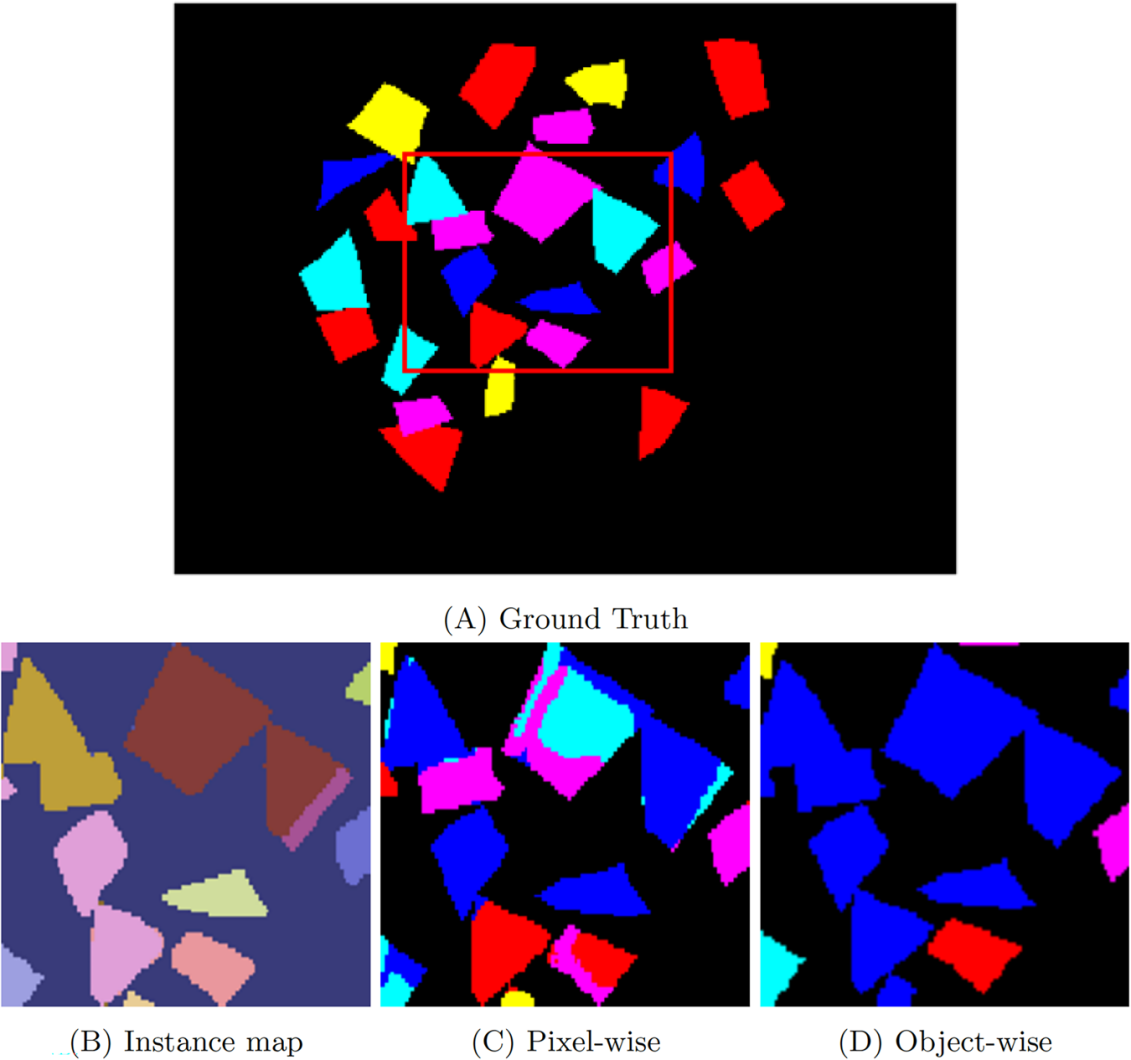
An example case where the zero-shot object-wise classification fails in sample 42, due to overlapping and touching pieces.

It is clear from Table 5 that object-wise classification via majority voting generally improves performance, but it fails in cases where the zero-shot SAM model misinterprets overlapping or touching objects as a single instance. This leads to incorrect class assignment during majority voting. An example is depicted in Fig. 8. Such limitations reflect the risk of directly applying large zero-shot models trained on diverse object datasets^39^ and highlight the need for the model’s fine-tuning on domain-specific annotations aligned with our object boundary definitions.

**Fig. 9:**
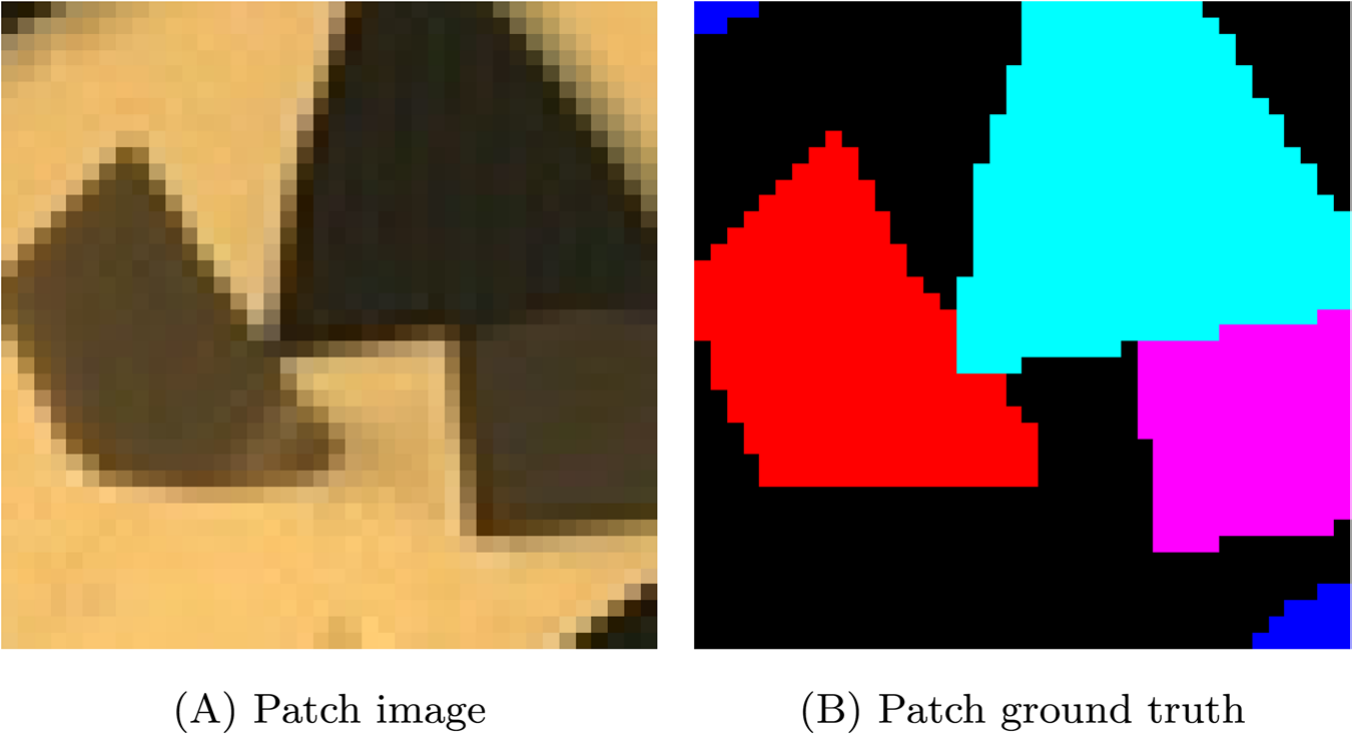
Confusing input for HSI Transformer encoders when more than one class/material appears in the input patch. The patch is taken from test sample 42 of Fig. 6.

At the pixel level, the Transformer models classify the center pixel of an HSI patch by treating each spectral band as a token. However, because the same spatial patch is repeated across all wavelengths, the tokens replicate similar spatial patterns, differing only in spectral intensity. This redundancy can overload the model with repeated patterns, encouraging overfitting and hindering convergence. Moreover, mixed-class patches exacerbate the problem: Fig. 9 shows an input patch extracted from sample 42 containing several classes, including Mesh, HTEL Anode, and HTEL Cathode spectra, where non-central pixel vectors from different classes are merged into the computation layers, degrading class learning and recognition. This underscores the importance of carefully selecting patch sizes to match object scale and minimize class mixing in close-range HSI.

Beyond modeling constraints, the dataset itself presents inherent material challenges. Electrolyzer components are high-tech materials with (a) dark-colored surfaces producing low-reflectance signals, (b) visually similar dark or gray tones across different classes, and (c) critical raw materials with masked or absent absorption features, leaving only general spectral patterns for the models to learn. These factors make Electrolyzers-HSI a benchmark for testing model robustness under difficult spectral conditions, but they also explain the relatively modest overall accuracies (OA pixel-wise  < 70%) observed even in pixel-level tasks. While the dataset may appear large in pixel count, HSI pixels are high-dimensional vectors (180 spectral bands after binning), and the limited number of physical samples restricts intra-class variability. As a result, labeled pixels are not fully independent, and the diversity of degradation states, contamination, and manufacturing differences remains underrepresented.

Based on the observed performance patterns, we outline several directions for improving and optimizing Transformer-based models for HSI classification:

- **Object Segmentation Fine-tuning**: Finetuning large zero-shot models to domain-specific boundaries.
- **Refined Input Tokenization**: Reduce redundancy through advanced spectral-spatial tokenization.
- **Architectural Enhancement**: Modify attention mechanisms to capture subtle spectral differences.
- **Data Engineering**: Acquire more diverse samples to reduce bias and improve generalization.

In terms of scope, Electrolyzers-HSI is the first dataset dedicated to electrolyzer materials and is therefore specific rather than broadly representative of all e-waste. Still, its acquisition methods, preprocessing pipeline, and modeling approaches are transferable to other close-range HSI tasks.

## Usage Notes

Direct visualization of the ground truth masks results in nearly black images, as pixel values within these masks range only from 0 to 5. While subtle variations corresponding to these low values may be visible upon close inspection, we recommend using the dedicated visualization function provided in the accompanying codebase to reproduce the mask representations shown in this study.

## Data Availability

The dataset is publicly available on the RODARE platform on the following page “https://rodare.hzdr.de/record/3668” and can be accessed freely.

## Code availability

The codes of this work were performed using Python and the PyTorch framework. The data processing and inference pipelines, together with the models’ weights, are available on “https://github.com/hifexplo/Electrolyzers-HSI”. The codes for the masking method with the zero-shot segmentation are available on: “https://github.com/hifexplo/Masking”. The ground truth masks were generated using “https://labelme.io/”. The used Crunchy preprocessing pipeline of raw hyperspectral data is “https://github.com/hifexplo/crunchy”. The training and inference codes from the validation models were deployed and modified from the original models repositories; MFT from: “https://github.com/AnkurDeria/MFT”, SpectralFormer and ViT from: “https://github.com/danfenghong/IEEE_TGRS_SpectralFormer”.
